# 5-Cyclo­hexyl-3-(4-fluoro­phenyl­sulfon­yl)-2-methyl-1-benzofuran

**DOI:** 10.1107/S1600536811006593

**Published:** 2011-03-02

**Authors:** Hong Dae Choi, Pil Ja Seo, Byeng Wha Son, Uk Lee

**Affiliations:** a Department of Chemistry, Dongeui University, San 24 Kaya-dong Busanjin-gu, Busan 614-714, Republic of Korea; b Department of Chemistry, Pukyong National University, 599-1 Daeyeon 3-dong, Nam-gu, Busan 608-737, Republic of Korea

## Abstract

In the title compound, C_21_H_21_FO_3_S, the cyclo­hexyl ring adopts a chair conformation. The 4-fluoro­phenyl ring makes a dihedral angle of 77.71 (4)° with the mean plane of the benzofuran fragment. In the crystal, mol­ecules are linked through inter­molecular C—H⋯O hydrogen bonds and aromatic π–π inter­actions between the furan rings of neighbouring mol­ecules [centroid–centroid distance = 3.578 (2) Å].

## Related literature

For the biological activity of benzofuran compounds, see: Aslam *et al.* (2006 ▶); Galal *et al.* (2009 ▶); Khan *et al.* (2005 ▶). For natural products with benzofuran rings, see: Akgul & Anil (2003 ▶); Soekamto *et al.* (2003 ▶). For our previous structural studies of related 3-(4-fluoro­phenyl­sulfon­yl)-2-methyl-1-benzo­furan derivatives, see: Choi *et al.* (2010**a* ▶,b* ▶).
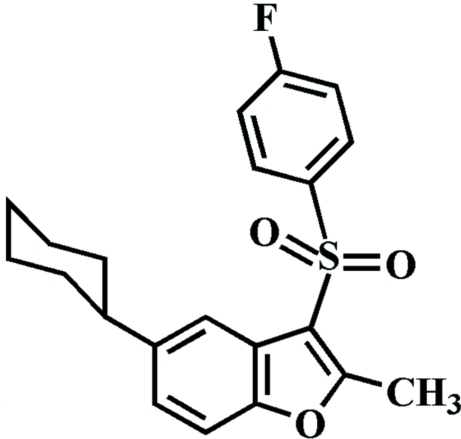


## Experimental

### 

#### Crystal data

C_21_H_21_FO_3_S
*M* *_r_* = 372.44
Triclinic, 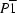

*a* = 9.2014 (3) Å
*b* = 10.2563 (4) Å
*c* = 11.1105 (4) Å
α = 80.564 (2)°
β = 66.317 (2)°
γ = 71.825 (2)°
*V* = 911.41 (6) Å^3^
*Z* = 2
Mo *K*α radiation
μ = 0.21 mm^−1^
*T* = 173 K
0.33 × 0.23 × 0.17 mm

#### Data collection

Bruker SMART APEXII CCD diffractometer
Absorption correction: multi-scan (*SADABS*; Bruker, 2009 ▶) *T*_min_ = 0.640, *T*_max_ = 0.746
15715 measured reflections
3967 independent reflections
3395 reflections with *I* > 2σ(*I*)
*R*_int_ = 0.034

#### Refinement

*R*[*F* ^2^ > 2σ(*F* ^2^)] = 0.039
*wR*(*F* ^2^) = 0.106
*S* = 1.03
3967 reflections
236 parameters
H-atom parameters constrained
Δρ_max_ = 0.24 e Å^−3^
Δρ_min_ = −0.39 e Å^−3^

### 

Data collection: *APEX2* (Bruker, 2009 ▶); cell refinement: *SAINT* (Bruker, 2009 ▶); data reduction: *SAINT*; program(s) used to solve structure: *SHELXS97* (Sheldrick, 2008 ▶); program(s) used to refine structure: *SHELXL97* (Sheldrick, 2008 ▶); molecular graphics: *ORTEP-3* (Farrugia, 1997 ▶) and *DIAMOND* (Brandenburg, 1998 ▶); software used to prepare material for publication: *SHELXL97*.

## Supplementary Material

Crystal structure: contains datablocks global, I. DOI: 10.1107/S1600536811006593/kp2311sup1.cif

Structure factors: contains datablocks I. DOI: 10.1107/S1600536811006593/kp2311Isup2.hkl

Additional supplementary materials:  crystallographic information; 3D view; checkCIF report

## Figures and Tables

**Table 1:** Hydrogen-bond geometry (Å, °)

*D*—H⋯*A*	*D*—H	H⋯*A*	*D*⋯*A*	*D*—H⋯*A*
C17—H17⋯O3^i^	0.93	2.59	3.2970 (19)	133
C20—H20⋯O1^ii^	0.93	2.60	3.285 (2)	131
C21—H21⋯O2^iii^	0.93	2.42	3.3114 (19)	160

Symmetry codes: (i) 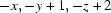; (ii) 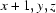; (iii) 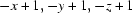.

**Fig. 1.**
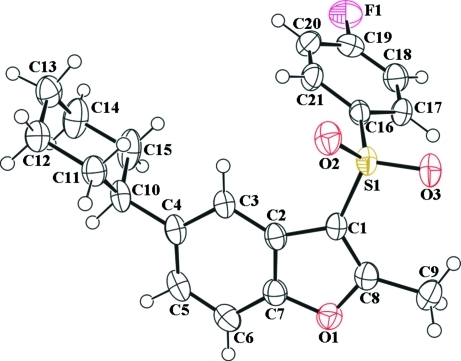
The molecular structure of the title compound with the atom numbering scheme. Displacement ellipsoids are drawn at the 50% probability level. H atoms are presented as a small spheres of arbitrary radius.

**Fig. 2.**
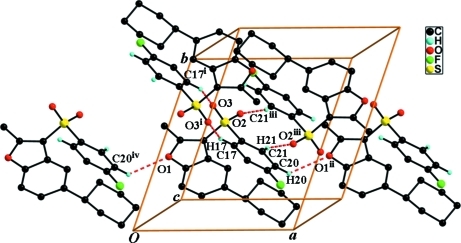
A view of the C–H···O hydrogen bonds (dotted lines) in the crystal structure of the title compound. [Symmetry codes: (i) - x, - y + 1, - z + 1; (ii) x + 1, y, z; (iii) - x+ 1, - y + 1, - z + 1; (iv) x - 1, y, z.]

**Fig. 3.**
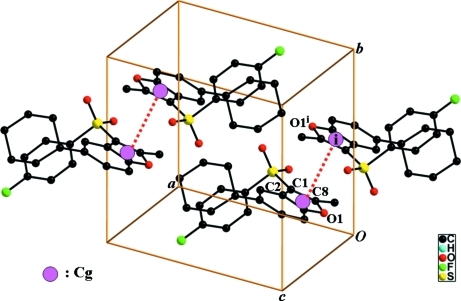
A view of the π–π interactions (dotted lines) in the crystal structure of the title compound. [Symmetry code: (i) - x, - y + 1, - z + 1.]

## supplementary crystallographic information

### Comment

Many compounds involving a benzofuran ring have attracted much attention owing to their interesting  pharmacological properties such as antifungal, antimicrobial, antitumor and antiviral activities (Aslam *et al.*, 2006; Galal *et al.*, 2009; Khan *et al.*, 2005). These compounds occur in a wide range of natural products (Akgul & Anil, 2003; Soekamto *et al.*, 2003). As a part of our ongoing program of the substituent effect on the solid state structures of 3-(4-fluorophenylsulfonyl)-2-methyl-1-benzofuran analogues (Choi*et al.*, 2010*a,b*), we report herein on the crystal structure of the title compound.

In the title molecule (Fig. 1), the benzofuran unit is essentially planar, with a mean deviation of 0.013 (1) Å from the least-squares plane defined by the nine constituent atoms. The cyclohexyl ring is in the chair conformation. The 4-fluorophenyl ring makes a dihedral angle of  77.71 (4)° with the mean plane of the benzofuran ring. The crystal packing (Fig. 2, Table 1) is stabilised by intermolecular C—H···O hydrogen bonds; the first one between a 4-fluorophenyl H atom and the oxygen of the O═S═O unit (Table 1; C17—H17···O3^i^), the second one between a 4-fluorophenyl H atom and the furan O atom (Table 1; C20—H20···O1^i^), the third one between a 4-fluorophenyl H atom and the oxygen of the O═S═O unit (Table 1; C21–H21···O2^iii^). The crystal packing (Fig. 3) is also stabilised by an aromatic π–π interaction between the furan rings of adjacent molecules, with a Cg···Cg^i^distance of 3.578 (2) Å (Cg is the centroid of the C1/C2/C7/O1/C8 furan ring).

### Experimental

3-Chloroperoxybenzoic acid, 77%  (426 mg, 1.9 mmol) was added in small portions to a stirred solution of 5-cyclohexyl-3-(4-fluorophenylsulfanyl)-2-methyl-1-benzofuran (306 mg, 0.9 mmol) in dichloromethane (40 mL) at 273 K. After being stirred at room temperature for 6 h, the mixture was washed with saturated sodium bicarbonate solution and the organic layer was separated, dried over magnesium sulfate, filtered and concentrated at reduced pressure. The residue was purified by column chromatography (hexane–ethyl acetate, 4:1 v/v) to afford the title compound as a colourless solid [yield 73%, m.p. 426–427 K; *R*_f_ = 0.68 (hexane–ethyl acetate, 2:1 v/v)]. Single crystals suitable for X-ray diffraction were prepared by slow evaporation of a solution of the title compound in benzene at room temperature.

### Refinement

All H atoms were positioned geometrically and refined using a riding model, with C–H = 0.93 Å  for aryl, 0.98 Å   for methine, 0.97 Å   for methylene and 0.96 Å   for methyl H atoms, respectively. *U*_iso_(H) =1.2*U*_eq_(C)  for aryl,  methine and methylene, and 1.5*U*_eq_(C) for methyl H atoms.

### Crystal data

**Table:**

C_21_H_21_FO_3_S	*Z* = 2
*M**_r_* = 372.44	*F*(000) = 392
Triclinic, *P*1	*D*_x_ = 1.357 Mg m^−^^3^
Hall symbol: -P 1	Mo *K*α radiation, λ = 0.71073 Å
*a* = 9.2014 (3) Å	Cell parameters from 6718 reflections
*b* = 10.2563 (4) Å	θ = 2.5–27.5°
*c* = 11.1105 (4) Å	µ = 0.21 mm^−^^1^
α = 80.564 (2)°	*T* = 173 K
β = 66.317 (2)°	Block, colourless
γ = 71.825 (2)°	0.33 × 0.23 × 0.17 mm
*V* = 911.41 (6) Å^3^	

### Data collection

**Table:**

Bruker SMART APEXII CCD diffractometer	3967 independent reflections
Radiation source: rotating anode	3395 reflections with *I* > 2σ(*I*)
graphite multilayer	*R*_int_ = 0.034
Detector resolution: 10.0 pixels mm^-1^	θ_max_ = 27.0°, θ_min_ = 2.0°
φ and ω scans	*h* = −11→11
Absorption correction: multi-scan (*SADABS*; Bruker, 2009)	*k* = −12→13
*T*_min_ = 0.640, *T*_max_ = 0.746	*l* = −14→14
15715 measured reflections	

### Refinement

**Table:**

Refinement on *F*^2^	Primary atom site location: structure-invariant direct methods
Least-squares matrix: full	Secondary atom site location: difference Fourier map
*R*[*F*^2^ > 2σ(*F*^2^)] = 0.039	Hydrogen site location: difference Fourier map
*wR*(*F*^2^) = 0.106	H-atom parameters constrained
*S* = 1.03	*w* = 1/[σ^2^(*F*_o_^2^) + (0.0513*P*)^2^ + 0.3006*P*] where *P* = (*F*_o_^2^ + 2*F*_c_^2^)/3
3967 reflections	(Δ/σ)_max_ = 0.001
236 parameters	Δρ_max_ = 0.24 e Å^−^^3^
0 restraints	Δρ_min_ = −0.39 e Å^−^^3^

### Special details

**Table:**

Geometry. All esds (except the esd in the dihedral angle between two l.s. planes) are estimated using the full covariance matrix. The cell esds are taken into account individually in the estimation of esds in distances, angles and torsion angles; correlations between esds in cell parameters are only used when they are defined by crystal symmetry. An approximate (isotropic) treatment of cell esds is used for estimating esds involving l.s. planes.
Refinement. Refinement of F^2^ against ALL reflections. The weighted R-factor wR and goodness of fit S are based on F^2^, conventional R-factors R are based on F, with F set to zero for negative F^2^. The threshold expression of F^2^ > 2sigma(F^2^) is used only for calculating R-factors(gt) etc. and is not relevant to the choice of reflections for refinement. R-factors based on F^2^ are statistically about twice as large as those based on F, and R- factors based on ALL data will be even larger.

### Fractional atomic coordinates and isotropic or equivalent isotropic displacement parameters (Å^2^)

**Table:**

	*x*	*y*	*z*	*U*_iso_*/*U*_eq_	
S1	0.17745 (5)	0.50397 (4)	0.70139 (3)	0.03247 (12)	
F1	0.60299 (14)	0.09412 (12)	0.93389 (12)	0.0614 (3)	
O1	−0.07799 (13)	0.32075 (11)	0.61095 (11)	0.0385 (3)	
O2	0.28066 (15)	0.57220 (12)	0.59295 (11)	0.0417 (3)	
O3	0.04350 (14)	0.58296 (11)	0.80566 (11)	0.0418 (3)	
C1	0.09922 (18)	0.40980 (15)	0.63832 (14)	0.0309 (3)	
C2	0.19131 (19)	0.32218 (15)	0.52695 (14)	0.0313 (3)	
C3	0.35461 (19)	0.28136 (15)	0.44039 (14)	0.0330 (3)	
H3	0.4340	0.3158	0.4458	0.040*	
C4	0.3973 (2)	0.18808 (16)	0.34550 (14)	0.0358 (3)	
C5	0.2739 (2)	0.14063 (17)	0.33786 (16)	0.0414 (4)	
H5	0.3033	0.0796	0.2734	0.050*	
C6	0.1113 (2)	0.18053 (18)	0.42166 (17)	0.0425 (4)	
H6	0.0307	0.1485	0.4152	0.051*	
C7	0.0751 (2)	0.27039 (16)	0.51537 (15)	0.0351 (3)	
C8	−0.06007 (19)	0.40433 (15)	0.68512 (15)	0.0345 (3)	
C9	−0.2136 (2)	0.46764 (19)	0.79404 (17)	0.0441 (4)	
H9A	−0.1905	0.5242	0.8411	0.066*	
H9B	−0.2558	0.3968	0.8528	0.066*	
H9C	−0.2941	0.5229	0.7588	0.066*	
C10	0.5742 (2)	0.13649 (16)	0.25294 (15)	0.0388 (4)	
H10	0.5752	0.0787	0.1905	0.047*	
C11	0.6477 (2)	0.25270 (16)	0.17308 (15)	0.0379 (4)	
H11A	0.5823	0.3044	0.1221	0.046*	
H11B	0.6428	0.3146	0.2327	0.046*	
C12	0.8261 (2)	0.19855 (17)	0.08055 (16)	0.0410 (4)	
H12A	0.8295	0.1467	0.0135	0.049*	
H12B	0.8707	0.2754	0.0371	0.049*	
C13	0.9320 (2)	0.10759 (18)	0.15317 (18)	0.0467 (4)	
H13A	0.9408	0.1624	0.2120	0.056*	
H13B	1.0420	0.0693	0.0903	0.056*	
C14	0.8602 (2)	−0.00825 (19)	0.23200 (19)	0.0583 (6)	
H14A	0.9268	−0.0608	0.2818	0.070*	
H14B	0.8633	−0.0693	0.1724	0.070*	
C15	0.6827 (3)	0.0469 (2)	0.32611 (18)	0.0578 (6)	
H15A	0.6810	0.1006	0.3911	0.069*	
H15B	0.6385	−0.0295	0.3720	0.069*	
C16	0.30614 (19)	0.38018 (15)	0.77139 (14)	0.0317 (3)	
C17	0.2384 (2)	0.33178 (17)	0.90047 (15)	0.0383 (4)	
H17	0.1261	0.3643	0.9490	0.046*	
C18	0.3384 (2)	0.23538 (19)	0.95594 (17)	0.0451 (4)	
H18	0.2956	0.2023	1.0423	0.054*	
C19	0.5031 (2)	0.18935 (18)	0.88040 (18)	0.0418 (4)	
C20	0.5731 (2)	0.23555 (18)	0.75333 (18)	0.0425 (4)	
H20	0.6853	0.2018	0.7054	0.051*	
C21	0.47341 (19)	0.33341 (17)	0.69787 (16)	0.0380 (4)	
H21	0.5179	0.3676	0.6121	0.046*	

### Atomic displacement parameters (Å^2^)

**Table:**

	*U*^11^	*U*^22^	*U*^33^	*U*^12^	*U*^13^	*U*^23^
S1	0.0357 (2)	0.0309 (2)	0.0242 (2)	−0.00993 (15)	−0.00282 (15)	−0.00406 (14)
F1	0.0580 (7)	0.0651 (7)	0.0675 (8)	−0.0096 (6)	−0.0381 (6)	0.0045 (6)
O1	0.0357 (6)	0.0430 (6)	0.0352 (6)	−0.0116 (5)	−0.0113 (5)	−0.0005 (5)
O2	0.0492 (7)	0.0392 (6)	0.0311 (6)	−0.0191 (5)	−0.0055 (5)	0.0024 (5)
O3	0.0423 (7)	0.0381 (6)	0.0338 (6)	−0.0055 (5)	−0.0030 (5)	−0.0123 (5)
C1	0.0320 (7)	0.0300 (7)	0.0244 (7)	−0.0058 (6)	−0.0061 (6)	−0.0017 (6)
C2	0.0383 (8)	0.0287 (7)	0.0234 (7)	−0.0077 (6)	−0.0100 (6)	0.0011 (6)
C3	0.0385 (8)	0.0319 (7)	0.0246 (7)	−0.0094 (6)	−0.0076 (6)	−0.0024 (6)
C4	0.0465 (9)	0.0311 (7)	0.0239 (7)	−0.0096 (7)	−0.0082 (6)	−0.0008 (6)
C5	0.0599 (11)	0.0370 (8)	0.0280 (8)	−0.0156 (8)	−0.0140 (7)	−0.0041 (7)
C6	0.0528 (10)	0.0434 (9)	0.0378 (9)	−0.0184 (8)	−0.0193 (8)	−0.0024 (7)
C7	0.0389 (8)	0.0353 (8)	0.0293 (8)	−0.0097 (6)	−0.0125 (6)	0.0020 (6)
C8	0.0371 (8)	0.0316 (7)	0.0293 (8)	−0.0065 (6)	−0.0103 (6)	0.0018 (6)
C9	0.0323 (8)	0.0485 (10)	0.0398 (9)	−0.0063 (7)	−0.0046 (7)	−0.0040 (7)
C10	0.0493 (9)	0.0333 (8)	0.0253 (7)	−0.0097 (7)	−0.0042 (7)	−0.0074 (6)
C11	0.0438 (9)	0.0334 (8)	0.0310 (8)	−0.0074 (7)	−0.0116 (7)	0.0008 (6)
C12	0.0445 (9)	0.0400 (9)	0.0321 (8)	−0.0111 (7)	−0.0086 (7)	−0.0006 (7)
C13	0.0452 (10)	0.0423 (9)	0.0433 (10)	0.0000 (8)	−0.0129 (8)	−0.0103 (8)
C14	0.0573 (12)	0.0407 (10)	0.0444 (11)	0.0086 (8)	−0.0046 (9)	0.0026 (8)
C15	0.0613 (12)	0.0428 (10)	0.0350 (9)	0.0066 (9)	−0.0022 (8)	0.0068 (8)
C16	0.0339 (8)	0.0348 (8)	0.0248 (7)	−0.0130 (6)	−0.0051 (6)	−0.0051 (6)
C17	0.0347 (8)	0.0483 (9)	0.0248 (7)	−0.0129 (7)	−0.0022 (6)	−0.0037 (7)
C18	0.0496 (10)	0.0547 (10)	0.0290 (8)	−0.0177 (8)	−0.0120 (7)	0.0031 (7)
C19	0.0451 (9)	0.0420 (9)	0.0468 (10)	−0.0128 (7)	−0.0247 (8)	−0.0027 (7)
C20	0.0301 (8)	0.0481 (10)	0.0458 (10)	−0.0118 (7)	−0.0071 (7)	−0.0090 (8)
C21	0.0351 (8)	0.0440 (9)	0.0293 (8)	−0.0160 (7)	−0.0014 (6)	−0.0041 (7)

### Geometric parameters (Å, °)

**Table:**

S1—O3	1.4336 (11)	C10—H10	0.9800
S1—O2	1.4360 (11)	C11—C12	1.524 (2)
S1—C1	1.7329 (16)	C11—H11A	0.9700
S1—C16	1.7599 (16)	C11—H11B	0.9700
F1—C19	1.3567 (19)	C12—C13	1.512 (2)
O1—C8	1.3675 (19)	C12—H12A	0.9700
O1—C7	1.3793 (19)	C12—H12B	0.9700
C1—C8	1.361 (2)	C13—C14	1.516 (3)
C1—C2	1.451 (2)	C13—H13A	0.9700
C2—C7	1.389 (2)	C13—H13B	0.9700
C2—C3	1.392 (2)	C14—C15	1.525 (2)
C3—C4	1.395 (2)	C14—H14A	0.9700
C3—H3	0.9300	C14—H14B	0.9700
C4—C5	1.403 (2)	C15—H15A	0.9700
C4—C10	1.511 (2)	C15—H15B	0.9700
C5—C6	1.378 (2)	C16—C21	1.387 (2)
C5—H5	0.9300	C16—C17	1.390 (2)
C6—C7	1.374 (2)	C17—C18	1.376 (2)
C6—H6	0.9300	C17—H17	0.9300
C8—C9	1.481 (2)	C18—C19	1.373 (2)
C9—H9A	0.9600	C18—H18	0.9300
C9—H9B	0.9600	C19—C20	1.369 (2)
C9—H9C	0.9600	C20—C21	1.381 (2)
C10—C15	1.529 (3)	C20—H20	0.9300
C10—C11	1.530 (2)	C21—H21	0.9300
			
O3—S1—O2	119.61 (7)	C12—C11—H11B	109.2
O3—S1—C1	108.35 (7)	C10—C11—H11B	109.2
O2—S1—C1	107.95 (7)	H11A—C11—H11B	107.9
O3—S1—C16	107.80 (7)	C13—C12—C11	111.76 (13)
O2—S1—C16	107.38 (7)	C13—C12—H12A	109.3
C1—S1—C16	104.78 (7)	C11—C12—H12A	109.3
C8—O1—C7	107.23 (12)	C13—C12—H12B	109.3
C8—C1—C2	107.66 (14)	C11—C12—H12B	109.3
C8—C1—S1	125.98 (12)	H12A—C12—H12B	107.9
C2—C1—S1	126.35 (12)	C12—C13—C14	111.51 (16)
C7—C2—C3	119.17 (14)	C12—C13—H13A	109.3
C7—C2—C1	104.49 (13)	C14—C13—H13A	109.3
C3—C2—C1	136.33 (15)	C12—C13—H13B	109.3
C2—C3—C4	118.99 (15)	C14—C13—H13B	109.3
C2—C3—H3	120.5	H13A—C13—H13B	108.0
C4—C3—H3	120.5	C13—C14—C15	111.21 (15)
C3—C4—C5	119.14 (15)	C13—C14—H14A	109.4
C3—C4—C10	120.85 (15)	C15—C14—H14A	109.4
C5—C4—C10	120.00 (14)	C13—C14—H14B	109.4
C6—C5—C4	122.90 (15)	C15—C14—H14B	109.4
C6—C5—H5	118.5	H14A—C14—H14B	108.0
C4—C5—H5	118.5	C14—C15—C10	111.44 (14)
C7—C6—C5	116.07 (16)	C14—C15—H15A	109.3
C7—C6—H6	122.0	C10—C15—H15A	109.3
C5—C6—H6	122.0	C14—C15—H15B	109.3
C6—C7—O1	125.83 (15)	C10—C15—H15B	109.3
C6—C7—C2	123.71 (15)	H15A—C15—H15B	108.0
O1—C7—C2	110.46 (13)	C21—C16—C17	121.00 (15)
C1—C8—O1	110.16 (13)	C21—C16—S1	119.73 (12)
C1—C8—C9	135.13 (15)	C17—C16—S1	119.26 (12)
O1—C8—C9	114.70 (14)	C18—C17—C16	119.55 (15)
C8—C9—H9A	109.5	C18—C17—H17	120.2
C8—C9—H9B	109.5	C16—C17—H17	120.2
H9A—C9—H9B	109.5	C19—C18—C17	118.27 (15)
C8—C9—H9C	109.5	C19—C18—H18	120.9
H9A—C9—H9C	109.5	C17—C18—H18	120.9
H9B—C9—H9C	109.5	F1—C19—C20	117.78 (16)
C4—C10—C15	111.52 (13)	F1—C19—C18	118.83 (16)
C4—C10—C11	112.73 (13)	C20—C19—C18	123.39 (16)
C15—C10—C11	110.17 (15)	C19—C20—C21	118.49 (15)
C4—C10—H10	107.4	C19—C20—H20	120.8
C15—C10—H10	107.4	C21—C20—H20	120.8
C11—C10—H10	107.4	C20—C21—C16	119.29 (15)
C12—C11—C10	111.90 (13)	C20—C21—H21	120.4
C12—C11—H11A	109.2	C16—C21—H21	120.4
C10—C11—H11A	109.2		
			
O3—S1—C1—C8	−4.02 (16)	C7—O1—C8—C9	−179.75 (13)
O2—S1—C1—C8	−134.92 (14)	C3—C4—C10—C15	67.17 (19)
C16—S1—C1—C8	110.87 (14)	C5—C4—C10—C15	−111.92 (18)
O3—S1—C1—C2	176.35 (12)	C3—C4—C10—C11	−57.37 (19)
O2—S1—C1—C2	45.45 (15)	C5—C4—C10—C11	123.54 (16)
C16—S1—C1—C2	−68.77 (14)	C4—C10—C11—C12	179.92 (14)
C8—C1—C2—C7	0.40 (16)	C15—C10—C11—C12	54.64 (18)
S1—C1—C2—C7	−179.91 (11)	C10—C11—C12—C13	−54.48 (19)
C8—C1—C2—C3	−178.10 (16)	C11—C12—C13—C14	54.41 (19)
S1—C1—C2—C3	1.6 (3)	C12—C13—C14—C15	−55.3 (2)
C7—C2—C3—C4	−0.8 (2)	C13—C14—C15—C10	56.3 (2)
C1—C2—C3—C4	177.49 (15)	C4—C10—C15—C14	178.45 (16)
C2—C3—C4—C5	1.5 (2)	C11—C10—C15—C14	−55.6 (2)
C2—C3—C4—C10	−177.55 (13)	O3—S1—C16—C21	−150.86 (13)
C3—C4—C5—C6	−1.0 (2)	O2—S1—C16—C21	−20.74 (15)
C10—C4—C5—C6	178.11 (15)	C1—S1—C16—C21	93.88 (13)
C4—C5—C6—C7	−0.3 (2)	O3—S1—C16—C17	28.49 (15)
C5—C6—C7—O1	−178.25 (14)	O2—S1—C16—C17	158.60 (13)
C5—C6—C7—C2	1.0 (2)	C1—S1—C16—C17	−86.78 (14)
C8—O1—C7—C6	178.84 (15)	C21—C16—C17—C18	−0.4 (2)
C8—O1—C7—C2	−0.53 (16)	S1—C16—C17—C18	−179.76 (13)
C3—C2—C7—C6	−0.5 (2)	C16—C17—C18—C19	−0.5 (3)
C1—C2—C7—C6	−179.30 (15)	C17—C18—C19—F1	−179.88 (15)
C3—C2—C7—O1	178.90 (12)	C17—C18—C19—C20	0.8 (3)
C1—C2—C7—O1	0.08 (16)	F1—C19—C20—C21	−179.41 (14)
C2—C1—C8—O1	−0.74 (17)	C18—C19—C20—C21	0.0 (3)
S1—C1—C8—O1	179.56 (10)	C19—C20—C21—C16	−0.9 (2)
C2—C1—C8—C9	179.96 (16)	C17—C16—C21—C20	1.1 (2)
S1—C1—C8—C9	0.3 (3)	S1—C16—C21—C20	−179.53 (12)
C7—O1—C8—C1	0.79 (16)		

### Hydrogen-bond geometry (Å, °)

**Table:**

*D*—H···*A*	*D*—H	H···*A*	*D*···*A*	*D*—H···*A*
C17—H17···O3^i^	0.93	2.59	3.2970 (19)	133
C20—H20···O1^ii^	0.93	2.60	3.285 (2)	131
C21—H21···O2^iii^	0.93	2.42	3.3114 (19)	160

Symmetry codes: (i) −*x*, −*y*+1, −*z*+2; (ii) *x*+1, *y*, *z*; (iii) −*x*+1, −*y*+1, −*z*+1.
